# Clinical Comparison of Conventional Testicular Sperm Extraction and Microdissection Techniques for Non-Obstructive Azoospermia

**DOI:** 10.4021/jocmr542w

**Published:** 2011-05-19

**Authors:** Ibrahim Fathi Ghalayini, Mohammed A. Al-Ghazo, Osama Bani Hani, Rami Al-Azab, Ibrahim Bani-Hani, Faheem Zayed, Yazan Haddad

**Affiliations:** a Urology Division, King Abdullah University Hospital, Jordan University of Science and Technology, Jordan; b Department of Obstetrics and Gynecology, King Abdullah University Hospital, Jordan University of Science and Technology, Jordan; c Princess Haya Biotechnology Center, Jordan University of Science and Technology, Jordan

## Abstract

**Background:**

We compared the efficacy of microdissection testicular sperm extraction (microdissection TESE) and conventional TESE in patients with non-obstructive azoospermia (NOA) and related the positive sperm recovery to certain variables: follicle-stimulating hormone (FSH) and luteinizing hormone (LH) levels, testicular volume and histology.

**Methods:**

Sperm retrieval rates (SRR) in patients with NOA who underwent microdissection TESE (n = 65) or conventional TESE (n = 68) were compared and related to the different variables.

**Results:**

SRR by microdissection TESE (56.9%) was significantly higher than conventional TESE (38.2%). There was a positive relation between the SRR and increased testicular volume or decreased FSH levels. No effect of Testosterone or Prolactin levels on SRR by using either technique was observed. Sperm were recovered from those with hypospermatogenesis in 84% and 92.9% by conventional and microdissection TESE, respectively (P = 0.3). In cases of maturation arrest the SRR was 27.3% and 36.4%, respectively (P = 0.6). In cases of Sertoli-cell-only syndrome (SCOS) the SRR was 6.2% and 26.9%, respectively (P = 0.03). No major operative complications occurred in any patient in either group, and no patient required post-operative hormone replacement to treat hypogonadism.

**Conclusions:**

Microdissection TESE significantly had twice better probability of success of SRR when compared to conventional TESE. No secure pre-operative prognostic elements of sperm recovery exist for NOA patients. Microdissection TESE appears to be recommendable in cases of atrophied testicles, high FSH concentration, or when SCOS with high FSH concentration can be predicted.

**Keywords:**

Microdissection TESE; Sperm retrieval; Non-obstructive azoospermia; Histopathology; FSH concentration; Orchidometry

## Introduction

Non-obstructive azoospermia (NOA) refers to absence of spermatozoa in semen analysis due to minimal or no production of fully developed spermatozoa in the testicles. Etiologies for testicular failure include genetic disorders such as sexual chromosomal abnormalities, translocations and microdeletions of the Y chromosome, cryptorchidism, testicular torsion, radiation and toxins [1-4].

Approximately 1% of all men and 10% of infertile men are affected by testicular failure as a result of NOA [5]. Testicular spermatozoa can be retrieved in some NOA men despite the absence of ejaculated spermatozoa in their semen, because of the existence of isolated foci of active spermatogenesis. Testicular spermatozoa can be retrieved successfully by the testicular sperm extraction (TESE) procedure and used for intracytoplasmic sperm injection (ICSI) in cases of NOA [6]. Such cases used to be treated with conventional TESE, including multiple biopsy samples of the testis. At present, in many centers this treatment has been replaced by microdissection TESE.

Direct vision with the operating microscope in microdissection TESE is of great advantage as larger, more opaque, whitish tubules, presumably containing more germ cells with active spermatogenesis, can be identified. This procedure is currently the best method for the certain identification of sperm, resulting in a high spermatozoa retrieval rate (SRR) and minimal postoperative complications. Histological findings are important in any comparison, since a relationship between SRR and testicular histopathology has been reported in the context of conventional TESE [5, 7, 8].

In our present study we compared SRR by microdissection TESE with that obtained by conventional multiple TESE in patients with histological findings including hypospermatogenesis, maturation arrest (MA), and Sertoli cell-only syndrome (SCOS). Diagnostic biopsy specimens were reviewed in all cases. As far as is known, there are few studies that compare conventional and microdissection TESE and relate the positive sperm recovery to certain variables, FSH and LH concentrations, testicular volume and testicular histology, which are all clinically relevant for NOA patients. We hope our study would add some information to support the previous reports.

## Materials and Methods

### Patients

A consecutive series of 133 men referred with a diagnosis of azoospermia were involved in the current study. All patients were diagnosed with NOA on the basis of a complete history, physical examination, endocrine profile, and chromosomal analysis before being scheduled for TESE with sperm freezing. Those with abnormal karyotyping were excluded from analysis. All patients underwent ejaculated semen examination at least 3 times before surgery. Patients also underwent careful evaluation by urologists concerning the duration of sterility, medical history, sexual function and results of a gynecologic evaluation of the spouse. Ultrasonography was performed to measure testicular volume and to determine the status of the epididymis and testis. Serum follicle-stimulating hormone (FSH) and luteinizing hormone (LH) were measured by immunoradiometric assay, while testosterone was measured by radioimmunoassay. Patients included in the study were allocated, according to the waiting list on the basis of the general operative theatre plan, after informed consent including explanations about results in the literature and invasiveness of the two procedures. Every operated testicle was classified according to the following variables: (i) testicular volume (V), categorized according to volume (≤ 8 ml, 9 - 12 ml, and ≥ 13 ml, i.e., normal); (ii) FSH concentration, categorized into two groups according to multiples of the normal range (N) (N and 2N: 1 - 24 mIU/mL, and > 3N: > 24 mIU/mL); (iii) testicular histology based on the most advanced pattern of spermatogenesis present such as hypospermatogenesis, maturation arrest (MA) and Sertoli cell-only syndrome (SCOS). Institutional Review Board (IRB) approval was obtained.

### Surgical approach

#### Conventional TESE

Conventional multiple TESE ordinarily was performed under general or local anesthesia. Through a small vertical incision in the median scrotal raphe, the skin, dartos muscle, and tunica vaginalis were opened to expose the tunica albuginea. The tunica albuginea was incised for about 4 mm at the upper pole near the head of the epididymis. If no sperm were seen in the initial sample, subsequent samples were taken from other locations, in the middle of the testis and at the lower pole opposite the rete testis, and subsequently from the contralateral testis. The procedure was terminated when sufficient spermatozoa were retrieved.

#### Microdissection TESE

Microdissection TESE was performed under general anesthesia according to the procedure reported previously [9, 10]. After the tunica albuginea was opened widely along the antimesenteric border, direct examination of the testicular parenchyma was performed under the operating microscope. An attempt was made to identify individual seminiferous tubules that were larger, more opaque and whiter than other tubules in the testicular parenchyma, which were considered to contain spermatozoa. The procedure was terminated when sperm were retrieved or further biopsy was thought likely to jeopardize the blood supply of the testis. If all tubules were seen to have an identical morphological appearance, at least three samples (upper, middle, and lower) that were equivalent to those from multiple TESE were obtained. The procedure was terminated when a sufficient volume of spermatozoa had been retrieved for ICSI. At the same time of testicular intervention in both procedures, a small tissue specimen was placed in Bouins solution and sent for histopathological examination.

### Sperm retrieval

Each sample was placed in a Petri dish filled with 0.5 ml of sperm wash media (Frederick-Maryland, USA), minced and shredded using sterile glass slides. Then, each sample was examined immediately by placing a small droplet of dispersed tissue suspension on the slide under a phase microscope using x 200 magnification for the presence of the testicular sperm. Further, all testicular samples for ICSI with 5 ml of media were subjected to centrifugation at 2000 x gravity for 10 minutes with careful, extended examination to determine the presence of even a single sperm after TESE procedure.

### Statistical analysis

The results were statistically evaluated using Mann-Whitney and Pearson’s Chi Square tests for comparison of the baseline data. A binary logistic regression was used to assess the adjusted odd ratios for the success of sperm retrieval in both methods of treatment. P-values less than 0.05 were considered statistically significant. All data were analyzed using the Statistical Package for the Social Sciences program (SPSS for Windows 16.0, SPSS Inc., USA).

## Results

### Patient background

Unexplained nonobstructive azoospermia was diagnosed in 68 men undergoing conventional and in 65 undergoing microdissection TESE. Two patients who underwent microdissection TESE had a history of intensive chemotherapy. Another 4 patients had bilateral undescended testes and 2 each underwent conventional and microdissection TESE. Pre-operative patient characteristics in the two groups including endocrine data and histopathological diagnosis were considered nearly identical (Table 1).

**Table 1:** A Comparison of Baseline Data Between Conventional Testicular Sperm Extraction (TESE) and Microdissection TESE Groups

	Conventional	Microdissection	P Value
Age	35.4 ± 7.1	34.8 ± 8.5	0.4
Mean FSH (IU/L)	16.7 ± 14.2	19.7 ± 12.5	0.06
FSH (IU/L), n (%)			0.13
< 24	52 (76.5%)	42 (64.6%)	
≥ 24	16 (23.5%)	23 (35.4%)	
LH (IU/L)	11.1 ± 8.0	11.0 ± 7.6	0.8
TEST (ng/ml)	3.9 ± 2.2	4.3 ± 2.2	0.3
PROL (ug/L)	8.7 ± 4.1	8.7 ± 2.9	0.4
Testicular volume (ml)	11.9 ± 4.6	11.8 ± 4.1	1.0
Patients with varicocele, n (%)	9 (13.2)	10 (15.4)	0.7
Patients after orchidopexy, n (%)	3 (4.4)	3 (4.6)	1.0
Histopathological diagnosis, n (%)			0.7
Hypospermatogenesis	25 (36.8)	28 (43.1)	
Maturation arrest	11 (16.2)	11 (16.9)	
Sertoli cell-only syndrome	32 (47.1)	26 (40.0)	

Testing by Mann Whitney and chi-square

### Success rate of sperm retrieval

Age did not have significant effect on sperm retrieval in either microdissection or conventional TESE. The success rate of sperm retrieval in patients with NOA was significantly higher for the microdissection than for the conventional TESE procedure (56.9% versus 38.2%, P = 0.03, Table 2). In 4 patients in whom bilaterally undescended testes were identified we could retrieve spermatozoa in the two patients who underwent the microdissection procedure and in one patient with the conventional TESE. Another 2 men who had undergone intensive chemotherapy for Lymphoma and testicular cancer underwent microdissection TESE. We succeeded in obtaining spermatozoa in the later case.

**Table 2:** Sperm Retrieval Rate According to the Class of Plasma FSH Concentration, Testicular Volume and Histopathological Diagnosis

		Conventional	Microdissection	P value
FSH (IU/L)	< 24	25/52 (48.1%)	28/42 (66.7%)	0.07
	≥ 24	1/16 (6.2%)	9/23 (39.1%)	0.02*
Testicular volume (ml)	≤ 8	3/18 (16.7%)	5/17 (29.4%)	0.4
	9 - 12	8/26 (30.8%)	15/27 (55.6%)	0.07
	> 12	15/24 (62.5%)	17/21 (81.0%)	0.1
Pathology	H	21/25 (84.0%)	26/28 (92.9%)	0.3
	MA	3/11 (27.3%)	4/11 (36.4%)	0.6
	SCOS	2/32 (6.2%)	7/26 (26.9%)	0.03*
Total		26/68 (38.2%)	37/65 (56.9%)	0.03*

H: hypospermatogenesis; MA: maturation arrest; SCOS: Sertoli cell-only syndrome; *: statistically significant

The influence of histological diagnosis on the success rate of sperm retrieval was considered. We obtained spermatozoa in 84% and 92.9% of those with the histological diagnosis of hypospermatogenesis, by conventional and microdissection TESE, respectively. We retrieved sperm in 27.3% and 36.4% of men with maturation arrest, respectively. For those with SCOS, SRR was significantly higher by microdissection TESE (26.9% versus 6.2%, P = 0.03, Table 3).

**Table 3:** Assessment of Success of Both Micro TESE and Conventional TESE Using Binary Logistic Regression

Factor	Microdissection			Conventional			Total		
	OR	CI (95%)	P-value	OR	CI (95%)	P-value	OR	CI (95%)	P-value
Age	1.01	0.96 - 1.08	0.67	0.97	0.90 - 1.04	0.37	0.99	0.95 - 1.04	0.73
Histopathology									
HS	1			1			1		
MA	0.04	0.01 - 0.29	0.001*	0.07	0.01 - 0.39	0.002*	0.06	0.02 - 0.21	< 0.001*
SCO	0.03	0.01 - 0.15	< 0.001*	0.01	0.002 - 0.08	< 0.001*	0.02	0.01 - 0.07	< 0.001*
Testicular volume	1.31	1.08 - 1.59	0.006*	1.24	1.07 - 1.43	0.004*	1.25	1.12 - 1.40	< 0.001*
Testicular volume range									
≤ 8	1			1			1		
9 - 12	3.00	0.83 - 10.90	0.10	2.22	0.50 - 9.89	0.30	2.59	0.99 - 6.74	0.05*
> 12	10.2	2.26 - 46.1	0.003*	8.33	1.88 - 37.0	0.005*	8.31	3.00 - 23.01	< 0.001*
FSH	0.96	0.92 - 1.00	0.05*	0.82	0.75 - 0.90	< 0.001*	0.93	0.90 - 0.96	< 0.001*
FSH range									
< 24	1			1			1		
≥ 24	0.32	0.11 - 0.92	0.04*	0.07	0.01 - 0.59	0.01	0.27	0.12 - 0.61	0.002*
LH	0.99	0.93 - 1.05	0.72	0.73	0.61 - 0.87	0.001*	0.92	0.87 - 0.97	0.003*
Testosterone	1.06	0.84 - 1.33	0.64	1.18	0.94 - 1.48	0.16	1.13	0.96 - 1.32	0.14
Prolactin	0.94	0.79 - 1.11	0.46	1.01	0.89 - 1.13	0.93	0.98	0.89 - 1.08	0.73

*: statistically significant

Sperm retrieval rate was positive in 10/39 (25.6%) patients with FSH ≥ 2N (24 mIU/mL) (9/23 with microdissection, 1/16 with conventional TESE); in 53/94 (56.4%) patients with < 2N (< 24 mIU/mL) (28/42 with microdissection, 25/52 with conventional TESE). SRR was significantly higher by microdissection TESE for those with FSH ≥ 2N (P = 0.02, Table 2). Increase in FSH levels showed significant failure of sperm retrieval in general (OR = 0.93, P < 0.001, Table 3). This failure was significant in conventional TESE by odds of 0.82 (P < 0.001) versus the odds of 0.96 by Microdissection TESE (P = 0.05). Also, increase in LH levels showed significant failure of sperm retrieval in general (OR = 0.93, P = 0.003, Table 3). This failure was also significant in conventional TESE (OR = 0.73, P < 0.001). However, LH levels had no significant influence on SRR by microdissection TESE (P = 0.72). There was no effect of Testosterone or Prolactin levels on SRR by using either technique.

**Table 4:** Assessment of Success of Microdissection TESE Compared to Conventional TESE Using Binary Logistic Regression

**Microdissection TESE Combined With Other Factors**	**OR**	**CI (95%)**	**P-value**
Alone	2.14	1.07 - 4.27	0.03*
Adjusted to FSH	3.54	1.55 - 8.08	0.003*
Adjusted to Testicular Volume	2.50	1.17 - 5.35	0.02*
Adjusted to Histopathology	2.96	1.10 - 7.99	0.03*
Adjusted to FSH and Testicular Volume	3.68	1.53 - 8.86	0.004*
Adjusted to FSH and Histopathology	3.47	1.25 - 9.60	0.01*
Adjusted to Testicular Volume and Histopathology	3.00	1.11 - 8.10	0.03*
Adjusted to FSH, Testicular Volume and Histopathology	3.56	1.28 - 9.92	0.02*

*: statistically significant

Increase in testicular volume significantly increased the success rate of sperm retrieval by 1.25 folds in both methods (P < 0.001, Table 3). This increase was significantly higher with the microdissection by 1.31 folds (P = 0.006) versus 1.24 folds with conventional TESE (P = 0.004). In Table 4 we assessed the success of microdissection compared to conventional TESE using binary logistic regression. The involvement of other factors in microdissection (FSH, testicular volume, histopathology, or any combination of them) significantly increases the success rate by a factor of 0.4 - 1.5.

According to the statistical analysis, FSH value and the surgical procedure were the two variables that could significantly predict positive sperm retrieval (P < 0.05). The testis volume and histology were shown to play a significant but less important role.

In addition, major postoperative complications, such as acute epididymitis, scrotal hematoma, and testicular hydrocele, were not seen significantly in this study. Only 5 (7.4%) patients from the conventional group and 3 (4.6%) patients from the microdissection group developed scrotal wall hematoma that resolved shortly during follow-up. In addition, no patient required hormone replacement therapy for treatment of post-operative hypogonadism.

## Discussion

The identification of areas in which spermatogenesis still occurs represents the background for the addition of magnification to TESE. Individual seminiferous tubules can be seen under the microscope allowing the identification of larger, whitish and opaque tubules in which spermatogenesis is active in opposition to tubules where no sperm production occurs [9]. This strategy could facilitate the removal of smaller amounts of testicular tissue, which becomes crucial in the presence of testicular atrophy. In addition, the identification of avascular regions for the opening of the tunica albuginea could minimize the chances of vascular injury. Multiple biopsy samples from different regions of the testis may increase the possibility of detecting spermatozoa with conventional TESE.

Some authors compared the results obtained by microdissection and conventional TESE in NOA patients [9-13] and reported a higher efficacy by microdissection in yielding positive sperm recovery, even when multiple TESE is performed [12, 13].

Several studies have compared the two techniques. Okada et al. [12] in their retrospective study including different patient groups, the SRR was 16.7% in the conventional TESE group and 44.6% in the microdissection group. Consistent with the results of most of the studies, the SRR in our study was significantly higher by microdissection TESE (56.9%) than conventional TESE (38.2%) (P = 0.03). Ramasamy et al. reported the outcomes of 460 patients with NOA treated with microdissection TESE [14]. In their report, the SRR by microdissection TESE was 57%, whereas that by conventional TESE was 32% which were approximately similar to our results. In a prospective comparative study of patients with NOA and bilaterally identical testicular histology who underwent conventional TESE on one testis and microdissection TESE on the other [11], the SRR by microdissection TESE was higher (47%) than by conventional TESE (30%) indicating the efficacy of microdissection TESE for sperm retrieval. In addition, postoperative acute and chronic complications were significantly lower in the microsurgical side compared with the conventional side [11].

We also investigated the SRR by microdissection TESE compared to that by conventional TESE of each type of testicular histology in patients with NOA. Furthermore, SRR by microdissection TESE for patients with hypospermatogenesis was 92.9%, whereas that by conventional TESE was 84%. We also found SRRs by microdissection TESE for maturation arrest and SCOS were 36.4% and 27.3%, respectively, whereas those by conventional TESE were 26.9%, and 6.2%. Only those of SCOS showed significantly higher SRR by microdissection TESE. Recently, Ramasamy et al. [14] reported excellent SRRs of 81%, 44%, and 41% for hypospermatogenesis, maturation arrest, and SCOS, respectively. The outcome of TESE may depend on factors other than urological technique, such as embryonic factors and human skills especially for those in the biology lab. In our study spermatozoa were detected in 61.7% of all cases during histopathogical examination of very small samples sent to the pathology lab compared to 45% detected by the people in the IVF lab. Thus, it is well accepted that microdissection TESE offers a great advantage for patients with all types of testicular histology. Tsujimura et al. [15] performed salvage microdissection TESE on the patients when conventional TESE failed to show spermatozoa and reported that the SRR increased with microdissection TESE.

Unlike these studies, there have been other studies showing no difference in the SRR between the two techniques [16]. There was no significant difference in the fertilization rates, between conventional TESE and microdissection TESE. Mulhall and co-workers [16] reported that the SRR was significantly higher with microdissection TESE than with conventional TESE in the patients with NOA and atrophic testis.

Microdissection TESE also avoided such complications as hematoma, fibrosis, and androgen decline, which otherwise might have been caused by conventional TESE in the patients with atrophic testes. We performed microdissection TESE on 17 patients whose testes were atrophic (testis volumes were ≤ 8 mL). Despite this effort, the SRR by microdissection was only 29.4% in this group which was still non-significantly higher than the conventional group (16.7%, P = 0.4). The small number of the cases may influence our statistics workup.

Clinically, testicular volume is correlated with spermatogenesis. Testicular volume had been found to have poor predictive value for successful TESE, however, because topographical variations in testicular pathology, independent of testicular volume, can occur [17]. Indeed, it had been reported that there is no statistically significant difference in testicular volume between patients with retrievable spermatozoa and those without [17, 18]. Furthermore, no lower limit of testicular volume for the absence of spermatozoa has been identified. Spermatozoa are often retrieved from testes with volumes less than 5 mL by microdissection TESE. Thus, small testicular volume itself does not preclude successful microdissection TESE [17]. Similar to others, we found a positive relation between the SRR and testis volume [19, 20].

Therefore, it can be suggested that the patients with NOA whose testis volumes are lower should be informed about the low SRR with conventional or microdissection TESE. The high amounts of removed testicular tissue may cause testicular insufficiency with a decrease in testosterone levels, especially in the hypoplastic/atrophic testicles. Schlegel [9] and Amer et al. [11] reported that the amount of removed testicular tissue in microdissectional TESE was significantly lower than with the conventional method. We could not measure the amount of testicular tissue removed in patients during the TESE operation. The missing information is a possible limitation of our study.

In our study, increase in FSH levels showed significant failure of sperm retrieval in general which was more significant in conventional TESE by odds of 0.82 (P < 0.001) versus the odds of 0.96 for microdissection TESE (P = 0.05). Although previous studies revealed a negative correlation between increased FSH levels and the SRR, recent studies showed no significant relation between FSH levels and the SRR [19]. Even in their study, Ramasamy and co-workers [21] reported lower SRR in the group of patients with FSH levels less than 15 IU/mL. Consistent with the literature, a significant relation between FSH levels and the SRR was detected in our study. In addition, testicular volume plays an important significant role in the SRR especially when the volume was greater than 12 cm (Table 3).

Regarding histopathology, only SCOS significantly affects the SRR of the two procedures. We and other authors found FSH value and the surgical procedure were the two variables significantly predicting positive sperm retrieval [20]. Increase in LH levels showed significant failure of sperm retrieval in general. Similar to others no difference was found in the LH and FSH levels between patients in whom sperm were retrieved successfully and those in whom no sperm were found [14].

The combination of different factors in microdissection TESE (FSH, testicular volume, histopathology, or any combination of them) significantly increased the success of SRR.

Microdissection TESE was reported to cause significantly fewer acute and chronic complications than conventional procedures based on post-operative ultrasonography [11]. However, major complications, such as acute epididymitis, big scrotal hematoma and testicular hydrocele were not seen in any patient in either the microdissection or the multiple TESE group in the present study. In this study, scrotal wall hematoma was non-significantly lower in the microdissection group compared to conventional TESE. In addition, no patient required hormone replacement therapy for treatment of post-operative hypogonadism. These findings suggest that microdissection TESE is safe in terms of both surgical and endocrinological complications.

### Conclusion

 Microdissection TESE significantly had twice better probability of success rate for sperm retrieval when compared with conventional TESE. All things considered, performing microdissection instead of conventional TESE is still the most effective treatment alternative in terms of high SRR and fewer complications. There was a relation between the SRR and testicular volume and FSH levels. Levels of testosterone or prolactin had no effect on the success rate of sperm retrieval using either method. Microdissection TESE appears to be recommendable especially in cases of atrophied testicles, high FSH concentration, or when SCOS with high FSH concentration can be predicted on the basis of the pre-operative prognostic data.
